# Isostructural rubidium and caesium 4-(3,5-di­nitro­pyrazol-4-yl)-3,5-di­nitro­pyrazolates: crystal engineering with polynitro energetic species

**DOI:** 10.1107/S2056989021010227

**Published:** 2021-10-13

**Authors:** Kostiantyn V. Domasevitch, Vira V. Ponomarova

**Affiliations:** a Inorganic Chemistry Department, National Taras Shevchenko University of Kyiv, Volodymyrska Str. 64/13, 01601 Kyiv, Ukraine

**Keywords:** crystal structure, rubidium, caesium, nitro­pyrazoles, energetic materials, hydrogen bonding

## Abstract

In the structures of the title salts, poly[[μ_4_-4-(3,5-di­nitro­pyrazol-4-yl)-3,5-di­nitro­pyrazol-1-ido]rubidium], [Rb(C_6_HN_8_O_8_)]_*n*_, (**1**), and its isostructural caesium analogue [Cs(C_6_HN_8_O_8_)_*n*_, (**2**), two independent cations *M*1 and *M*2 (*M* = Rb, Cs) are situated on a crystallographic twofold axis and on a center of inversion, respectively. Mutual inter­molecular hydrogen bonding between the conjugate 3,5-dinito­pyrazole NH-donor and 3,5-di­nitro­pyrazole N-acceptor sites of the anions [N⋯N = 2.785 (2) Å for (**1**) and 2.832 (3) Å for (**2**)] governs the self-assembly of the translation-related anions in a predictable fashion. Such one-component modular construction of the organic subtopology supports the utility of the crystal-engineering approach towards designing the structures of polynitro energetic materials. The anionic chains are further linked by multiple ion–dipole inter­actions involving the 12-coordinate cations bonded to two pyrazole N-atoms [Rb—N = 3.1285 (16), 3.2261 (16) Å; Cs—N = 3.369 (2), 3.401 (2) Å] and all of the eight nitro O-atoms [Rb—O = 2.8543 (15)–3.6985 (16) Å; Cs—O = 3.071 (2)–3.811 (2) Å]. The resulting ionic networks follow the CsCl topological archetype, with either metal or organic ions residing in an environment of eight counter-ions. Weak lone pair–π-hole inter­actions [pyrazole-N atoms to NO_2_ groups; N⋯N = 2.990 (3)–3.198 (3) Å] are also relevant to the packing. The Hirshfeld surfaces and percentage two-dimensional fingerprint plots for (**1**) and (**2**) are described.

## Abstract 2

In the structures of the title salts, two independent cations (Rb, Cs) are situated on a crystallographic twofold axis and on a center of inversion, respectively. Mutual inter­molecular hydrogen bonding between the conjugate 3,5-dinito­pyrazole NH-donor and 3,5-di­nitro­pyrazolate N-acceptor sites of the anions governs the self-assembly of the translation-related anions in a predictable fashion. The anionic chains are further linked by multiple ion–dipole inter­actions involving the 12-coordinate cations bonded to two pyrazole N-atoms and all of the eight nitro O-atoms. The resulting ionic networks follow the CsCl topological archetype, with either metal or organic ions residing in an environment of eight counter-ions. Weak lone pair–π-hole inter­actions are also relevant to the packing.

## Chemical context

Many issues of crystal engineering, in regard to control over bonding patterns, supra­molecular topologies, mol­ecular packing, and crystal morphologies are highly relevant to the area of energetic materials. In particular, non-covalent contacts involving common explosophore nitro groups (Bauzá *et al.*, 2017 ▸) establish pathways to transmit inter­molecular inter­actions and they are often responsible for higher densities of the solids (Zhang *et al.*, 2000 ▸). The layered architectures of the energetic solids provide better buffering against external mechanical stimuli, which is essential for developing insens­itive materials (Zhang *et al.*, 2008 ▸). At the same time, incorp­oration of specific coordination geometries for the assembly of metal–organic solids offers potential for the synthesis of new perchlorate-free flame colorants and pyrotechnics (Glück *et al.*, 2017 ▸). However, successful applications of the crystal-engineering methodology toward designing the structures of polynitro compounds are relatively rare, so far (Domasevitch *et al.*, 2020 ▸). This is predetermined by a lack of reliable supra­molecular synthons comprising the nitro groups, which are only weak acceptors of conventional hydrogen bonds (Robinson *et al.*, 2000 ▸) and are only weak donors with respect to the metal ions. A more severe limitation is associated with the need for direct bonding between the nitro-rich functionalities only, since the incorporation of any low-energetic component or solvent mol­ecules is an inevitable penalty to the performance. Such dilution of the energetic moieties in the crystals is relevant, for example, to a series of hydrogen-bonded solids prepared by Aakeröy *et al.* (2015 ▸) with acidic ethyl­enedinitramine and common bitopic pyridine-N bases. 
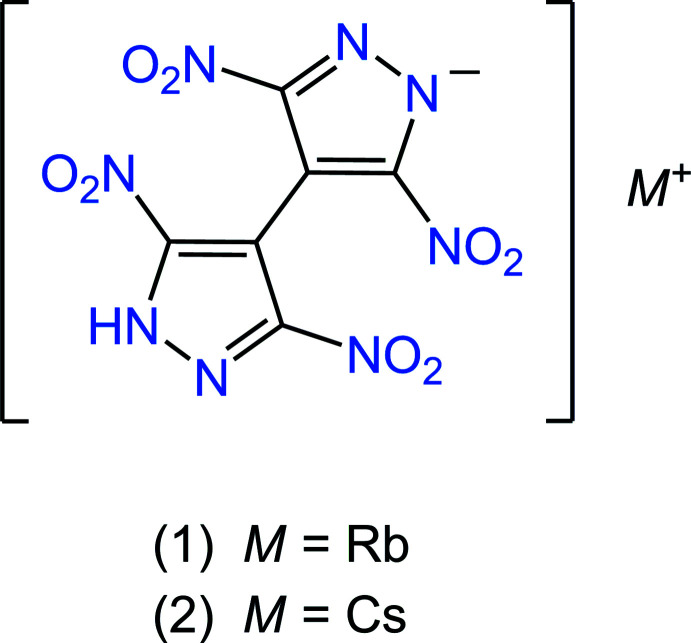


Recently, we have reported a new strategy for the construction of energetic salts, which offers higher degree of control over the structure. Double functionality of the well-performing material 3,3′,5,5′-tetra­nitro-4,4′-bi­pyrazole [H_2_(TNBP)] (Domasevitch *et al.*, 2019 ▸) grants synthetic access either to singly or doubly anionic species [{H(TNBP)}^−^ and {TNBP}^2−^, respectively]. The former combine conjugate di­nitro­pyrazole donor and di­nitro­pyrazolate acceptor sites for sustaining particularly strong N—H⋯N bonding. In fact, such bonding of two explosophores dominated the self-assembly in a very predictable fashion and it was traced in all of the previously examined salts with a range of nitro­gen-rich cations (Gospodinov *et al.*, 2020 ▸). That the resulting networks are ionic may find further applications to the synthesis of inorganic nitro-rich salts, based upon Li^+^, Rb^+^, Cs^+^, Sr^2+^, Ba^2+^ and other *s*- and *p*-block cations, which are a new generation of ‘green’ pyrotechnic formulations (Steinhauser & Klapötke, 2008 ▸).

Following the above findings, we now describe the synthesis and structure of rubidium and caesium 4-(3,5-di­nitro­pyrazol-4-yl)-3,5-di­nitro­pyrazolates *M*{H(TNBP)} [*M* = Rb (1) and Cs (2)], incorporating the peculiar half-deprotonated bi­pyrazole tectons. These materials may give an insight into the development of flame colorants in pyrotechnics: rubidium and caesium compounds exhibit, respectively, purple and orange colors when burned.

## Structural commentary

**Figure 1:**
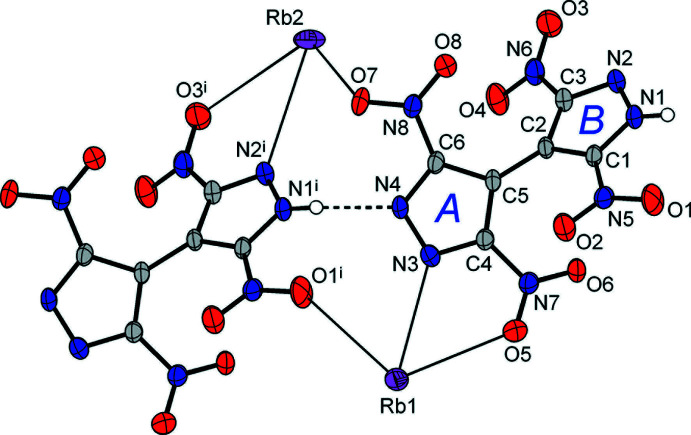
The mol­ecular structure and the atom-labeling scheme for (**1**) [the atom labeling for (**2**) is identical, with Cs1 and Cs2 instead of Rb1 and Rb2], with displacement ellipsoids drawn at the 50% probability level and the N—H⋯N hydrogen bond shown as a dashed line. [Symmetry code: (i) *x*, *y* + 1, *z*.]

The title compounds are isostructural, crystallizing in space group *C*2/*c*. The mol­ecular structure of the rubidium salt (**1**) is shown in Fig. 1 ▸, with the unique part comprising one organic anion {H(TNBP)}^−^ (or C_6_HN_8_O_8_^−^) and two cations situated on a crystallographic twofold axis [Rb1] or on a center of inversion [Rb2]. The easy formation of such salts is con­ditioned by the appreciable acidity of polynitro­pyrazoles, *c.f.* p*K*_a_ = 3.14 for 3,5-di­nitro­pyrazole *versus* 14.63 for the parent pyrazole (Janssen *et al.*, 1973 ▸), while for the crystallization of singly charged hydrogen bipyrazolate derivatives, the weakly polarizing, large Rb^+^ and Cs^+^ cations are important.

**Figure 2:**
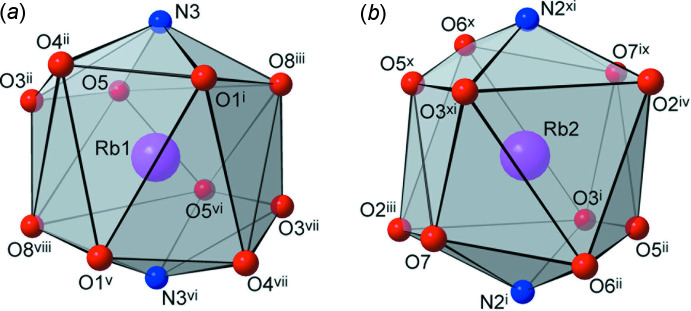
Twelvefold coordination environments adopted by the Rb1 and Rb2 ions in (**1**), in the form of distorted icosa­hedra. The coordination of the two Cs ions in (**2**) is almost identical. [Symmetry codes: (i) *x*, *y* + 1, *z*; (ii) −*x* + 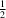 , *y* + 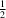 , −*z* + 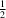 ; (iii) −*x* + 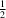 , −*y* + 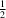 , −*z*; (iv) *x* − 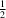 , *y* + 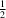 , *z*; (v) −*x* + 1, *y* + 1, −*z* + 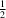 ; (vi) −*x* + 1, *y*, −*z* + 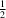 ; (vii) *x* + 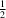 , *y* + 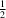 , *z*; (viii) *x* + 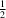 , −*y* + 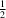 , *z* + 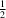 ; (ix) −*x*, −*y* + 1, −*z*; (*x*) *x* − 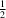 , −*y* + 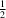 , *z* − 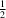 ; (xi) −*x*, −*y*, −*z*.]

**Figure 3:**
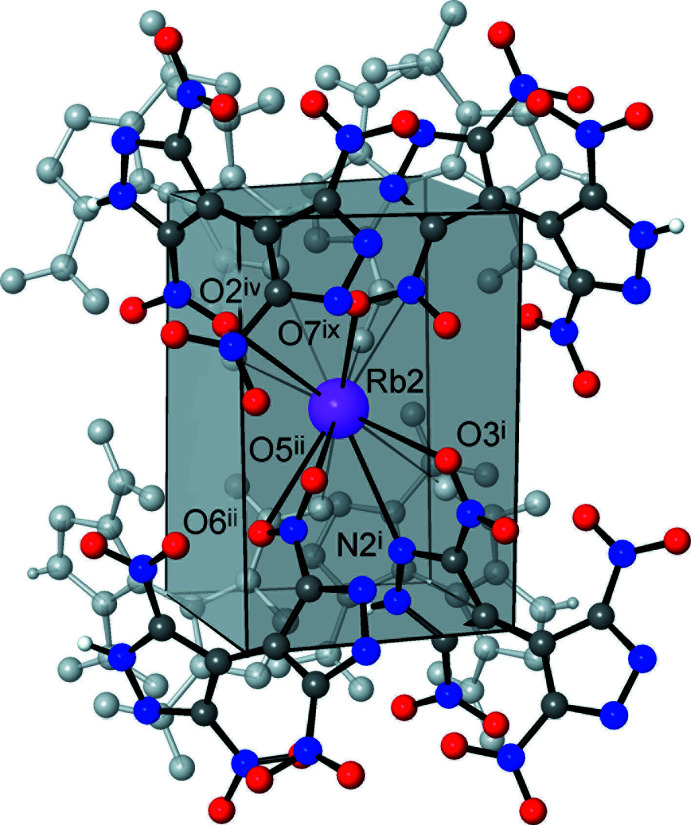
The Rb2 ion resides inside a supra­molecular prism (represented here as a gray box) adopted by eight anions, which complete the coordination environment. The vertices of the prism are built through the mid-points of the central C—C bonds of the mol­ecules. The environments of Rb1 and the respective Cs ions in the structure of (**2**) are similar. [Symmetry codes: (i) *x*, *y* + 1, *z*; (ii) −*x* + 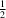 , *y* + 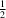 , −*z* + 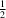 ; (iv) *x* − 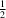 , *y* + 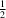 , *z*; (ix) −*x*, −*y* + 1, −*z*.]

Both unique metal ions exhibit exceptionally high coord­in­ation numbers of twelve, which are completed with ten O atoms [Rb—O = 2.8543 (15)–3.6985 (16) Å; Cs—O 3.071 (2)–3.811 (2) Å] and two N atoms of the pyrazole rings [Rb—N = 3.1285 (16) and 3.2261 (16) Å; Cs—N = 3.369 (2) and 3.401 (2) Å] (Tables 1 ▸ and 2 ▸). Most of these separations slightly exceed the sum of the corresponding ionic radii [which are *M*—O = 3.13 and 3.28 Å; *M*—N = 3.18 and 3.34 Å for 12-coordinate Rb and Cs ions, respectively (Shannon, 1976 ▸)], indicating the weakness of these relatively distal ion–dipole inter­actions. This may be best related to the bonding in the ionic salts with polynitro anions lacking conventional donor sites. For example, in caesium picrate, the cations display a comparable 12-fold coordination and a wide spread of Cs—O separations of 3.028 (3)–3.847 (2) Å (Schouten *et al.*, 1990 ▸). The coordination polyhedra of the two unique cations are very similar and represent essentially distorted icosa­hedra (Fig. 2 ▸). These are completed with a twofold axis [for *M*1] or inversion [for *M*2] related pairs of chelating nitro­pyrazole-N,O groups, pseudo-chelating NO_2_ groups and two singly coordinated NO_2_ groups. Both kinds of cations reside in a closest environment of eight {H(TNBP)}^−^ anions, which maintain supra­molecular boxes with a small inter­nal cavity for the cation (Fig. 3 ▸). It is notable that all of the eight O atoms present and the two pyrazole N atoms coordinate to the metal ions.

The main geometrical parameters of the organic anions are very similar to those of the parent [H_2_(TNBP)] (Domasevitch *et al.*, 2019 ▸). In the case of (**1**), the protolytic inequivalency of two pyrazole halves is reflected by the ring C—N distances, which are almost the same for anionic ring *A* (atoms C4/C5/C6/N3/N4) [N3—C4 = 1.343 (2) and N4—C6 1.348 (2) Å] and are slightly differentiated for the neutral ring *B* (C1/C2/C3/N1/N2) [N1—C1 1.348 (2) and N2—C3 1.331 (2) Å] (Fig. 1 ▸). In addition, the deprotonation causes slight elongation of the N—N bond, which is 1.336 (2) Å for ring *B* and 1.347 (2) Å for ring *A*. Even more sensitive parameters are the bond angles at the N atoms, which are perceptibly different for the former fragment [N2—N1—C1 = 110.67 (15); C3—N2—N1 = 104.29 (15)°], being much closer for the latter [106.38 (15) and 107.59 (15)°]. In the case of (**2**), the corresponding geometries are nearly identical for rings *A* and *B* [C—N = 1.340 (3)–1.346 (3) Å; N2—N1—C1 = 109.8 (2); N3—N4—C6 = 109.6 (2)° and N1—N2—C3 = 104.9 (2); N4—N3—C4 = 105.1 (2)°]. This situation agrees with the disorder of the H atoms between two positions [at the N1 or N4 carrier atoms] within the N—H⋯N hydrogen bond in (**2**) as discussed below.

In both structures, the {H(TNBP)}^−^ anions display twisted conformations, with the dihedral angles between the rings being 42.99 (8) and 44.86 (10)° for (**1**) and (**2**), respectively. These angles, however, are unusually small. For example, typical parameters for the structurally similar 3,3′,5,5′-tetra­methyl-4,4′-bi­pyrazole unit are 65–90° (Ponomarova *et al.*, 2013 ▸). The flattening of the {H(TNBP)}^−^ anion suggests certain attractivity in steric inter­actions of the NO_2_ groups, which generates a set of short intra­molecular O⋯N contacts, the shortest being O2⋯N7 at 2.786 (3) Å observed for (**2**). Indeed, the nitro/nitro stackings are energetically favorable, as a special kind of lone pair–π-hole bond (Bauzá *et al.*, 2017 ▸).

## Supra­molecular features

The ionic structures of the title compounds may be regarded as three-dimensional networks, which are related to the structure of CsCl. The metal ions themselves constitute a distorted primitive cubic framework with the cells representing elongated prisms [the *M*⋯*M* edges are 5.2560 (3), 6.5962 (3), 8.8395 (8) and 5.4775 (4), 6.3932 (5), 9.1482 (12) Å for (**1**) and (**2**), respectively]. Every such cell is populated with the organic anion and, conversely, every cation resides inside the distorted prismatic box of eight anions (Figs. 3 ▸ and 4 ▸).

**Figure 4:**
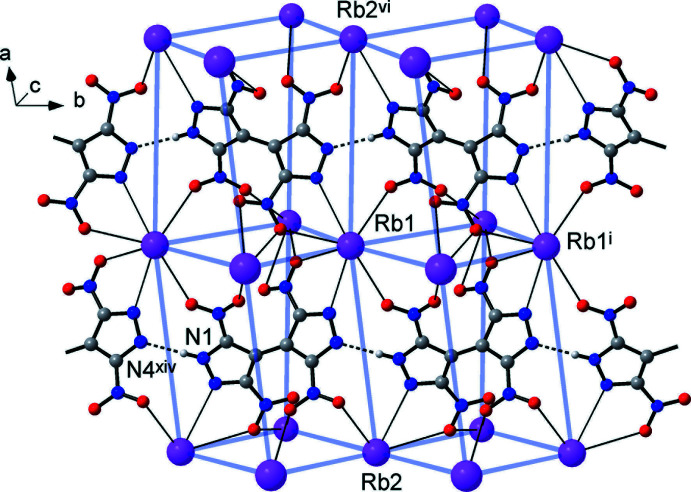
Fragment of the crystal structure of (**1**), showing the polar hydrogen-bonded anionic chains propagating along the *b*-axis direction, in the environment of the Rb cations. Blue lines indicate a pseudo-primitive cubic net arrangement of the cations, with every cell populated by a single anion (*c.f.* the structure of CsCl). [Symmetry codes: (i) *x*, *y* + 1, *z*; (vi) −*x* + 1, *y*, −*z* + 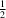 ; (xiv) *x*, *y* − 1, *z*.]

Beyond Coulombic attraction, the principal supra­molecular inter­action is strong and directional N—H⋯N hydrogen bonding between the pyrazole and pyrazolate halves of translation-related anions [N1⋯N4^xiv^ = 2.785 (2) and 2.832 (3) Å; H⋯N^xiv^ = 1.93 and 1.99 Å; N1*H*⋯N4^xiv^ = 166 and 163° for (**1**) and (**2**), respectively; symmetry code: (xiv) *x*, *y* − 1, *z*], arranging the latter into linear polar chains propagating along the *b*-axis direction (Fig. 4 ▸). Such bonding involving the conjugate acid (pyrazole-NH) and base (pyrazolate-N) sites is a very rare, if not the only, example of a highly reliable supra­molecular synthon for crystal engineering with energetic polynitro derivatives. In fact, the conjugate inter­actions are relevant for many organic species, *e.g*. carboxyl­ates (Speakman, 1972 ▸) and oximes (Domasevitch *et al.*, 1998 ▸), being often the most crucial bonding for the crystal patterns. With the aid of such a synthon, the assembly of the organic subtopology of lower dimensionality is possible in a very rational and predictable fashion and the title structures exactly follow the motifs of previously examined NH_3_OH^+^ and 3,3′,5,5′-tetra­methyl-4,4′-bipyrazolium {H(TNBP)}^−^ salts (Gospodinov *et al.*, 2020 ▸).

**Figure 5:**
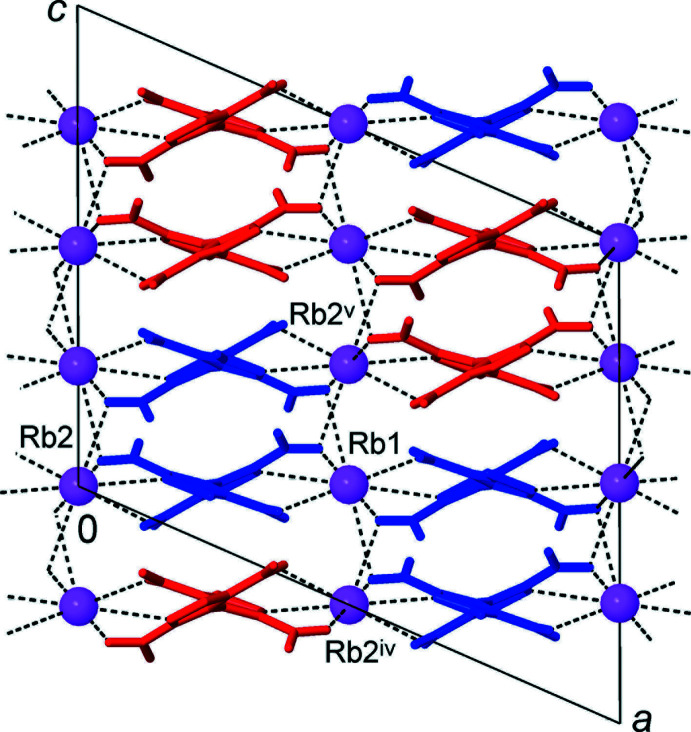
Structure of (**1**) viewed in projection on the *ac* plane (down the direction of the anionic chains) showing the organic layers, which are separated by layers of the cations. The chains of opposite polarity are identified by blue and red colors. [Symmetry codes: (iv) *x* − 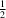 , *y* + 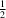 , *z*; (v) −*x* + 1, *y* + 1, −*z* + 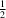 .]

**Figure 6:**
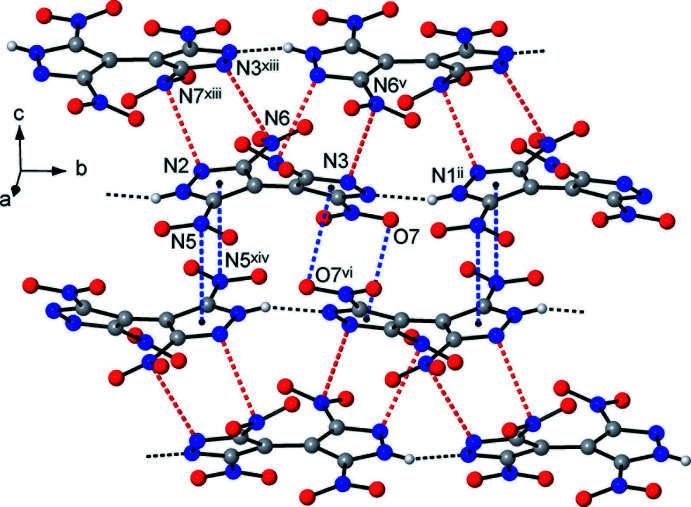
A suite of non-covalent inter­actions of the {H(TNBP)}^−^ anions, with two kinds of lone pair–π-hole bonds (marked in red) and two kinds of nitro/pyrazole stacks (marked in blue) complementing the conventional hydrogen bonding. [Symmetry codes: (ii) −*x* + 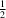 , *y* + 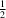 , −*z* + 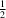 ; (v) −*x* + 1, *y* + 1, −*z* + 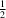 ; (vi) −*x* + 1, *y*, −*z* + 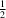 ; (xiii) −*x* + 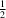 , −*y* − 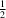 , −*z*; (xiv) *x*, *y* − 1, *z*.]

The above hydrogen-bonded chains associate to yield layers lying parallel to the *ac* plane and the latter are separated by the layers of metal cations (Fig. 5 ▸). There are two kinds of weaker inter­actions, which facilitate close packing of the chains. The first of these is identified by close N3⋯N6^ii^ and N2⋯N7^xii^ contacts [the shortest of 2.990 (3) Å] originating in situation of the pyrazole N atoms almost exactly above the NO_2_ N atoms (Table 3 ▸). This peculiar lone pair–π-hole inter­action occurs instead of the more common NO_2_/NO_2_ bonding (Bauzá *et al.*, 2017 ▸), which is also relevant for the structure of [H_2_(TNBP)] itself (Domasevitch *et al.*, 2019 ▸). One can note that extensive ion-dipole inter­actions *M*⋯O_2_N in (**1**) and (**2**) mitigate against mutual inter­actions of nitro groups, which are totally eliminated from the suite of supra­molecular bonds. The second type of inter­chain inter­action is stacking between pairs of inversion-related pyrazole and pyrazolate rings (Fig. 6 ▸), with the O7 and N5 atoms situated nearly above the centroids of the rings *A* ^iii^ and *B* ^xiii^, respectively [symmetry codes: (iii) −*x* + 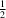 , −*y* + 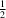 , −*z*; (xiii) −*x* + 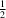 , −*y* − 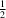 , −*z*.] (Table 4 ▸). As a result of the inversion symmetry of the stacks, the alignment of two polar hydrogen-bonded chains in (**1**) is anti­parallel, while the above lone pair–π-hole inter­actions support coherent alignment of the contributing chains (Fig. 6 ▸). This results in pairing of the chains possessing identical polarities (Fig. 5 ▸). In the structure of (**2**), the polarity of the chains is eliminated because of the disorder of the H atoms in the N—H⋯N/N⋯H—N bonds.

## Hirshfeld analysis

**Figure 7:**
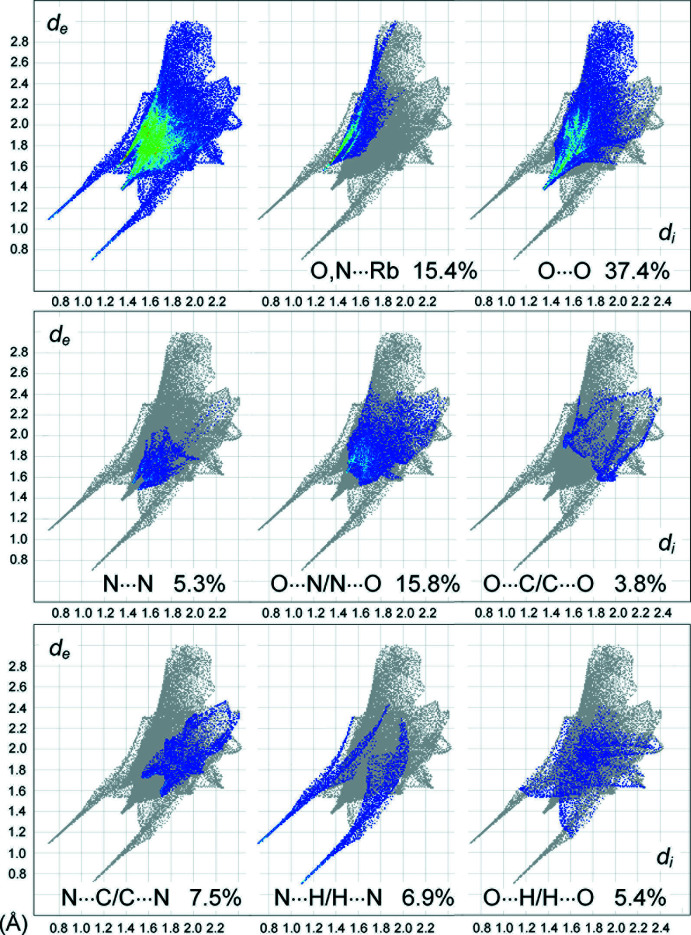
Two-dimensional fingerprint plots for the individual anions in (**1**), and delineated into the principal contributions of O,N⋯Rb, O⋯O, N⋯N, O⋯N/N⋯O, O⋯C/C⋯O, N⋯C/C⋯C, N⋯H/H⋯N and O⋯H/H⋯O contacts. Other contacts are C⋯H/H⋯C (1.5%) and C⋯C (1.0%).

The supra­molecular inter­actions in the title structures were also assessed by Hirshfeld surface analysis (Spackman & Byrom, 1997 ▸; McKinnon *et al.*, 2004 ▸; Hirshfeld, 1977 ▸; Spackman & McKinnon, 2002 ▸) performed with *CrystalExplorer17* (Turner *et al.*, 2017 ▸). The contributions of different kinds of inter­atomic contacts to the Hirshfeld surfaces of the individual anions are listed in Table 5 ▸ and the fingerprint plots for (**1**) are shown in Fig. 7 ▸. The most significant contributors are O⋯O contacts (37.4%), while the fraction of O,N⋯Rb (15.4%) is relatively modest due to the larger lengths of the ion–dipole inter­actions. The shortest O⋯O separation on the plot of ∼2.8 Å corresponds to the contact O1⋯O8^xiii^ = 2.741 (2) Å [2.732 (3) Å in (**2**); symmetry code (xiii) −*x* + 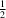 , −*y* − 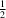 , −*z*]. We note that slight contraction of the O⋯*M* fraction in the case of *M* = Rb [13.6% for (**1**) and 13.0% for (**2**)] coincides with a larger contribution of less favorable O⋯O contacts [37.4% for (**1**) and 35.5% for (**2**)]. This may be an additional factor destabilizing the structure: the crystals of (**1**) eventually decompose under the mother solution, unlike the stable Cs analogue. The lone pair–π-hole pyrazole-NO_2_ inter­actions generate 5.3% (**1**) and 6.3% (**2**) of the contacts of the Hirshfeld surfaces, with the shortest N⋯N = 2.9 Å. The nature of the O⋯N/N⋯O and N⋯C/C⋯N contacts [in total 23.3% (**1**) and 22.8% (**2**)] is similar, since they correspond to the stacking of pyrazole and NO_2_ groups with shortest O⋯N and N⋯C distances of 3.2 and 3.3 Å, respectively. However, there are no pairs of the features that are characteristic for the mutual O⋯N/N⋯O inter­actions of NO_2_ groups themselves (Domasevitch *et al.*, 2020 ▸). The contributions of the O⋯H/H⋯O and N⋯H/H⋯N contacts are comparable and perceptible [5.4 and 6.9% for (**1**) and 5.2 and 6.6% for (**2**)], but only the latter correspond to hydrogen bonding, as is reflected in the plots. These bonds are responsible for a pair of very sharp features pointing to the lower left, with a shortest contact of 1.9 Å, whereas O⋯H/H⋯O contacts are identified only with a diffuse collection of points between the above features and with a shortest contact of 2.8 Å.

## Synthesis and crystallization

3,3′,5,5′-Tetra­nitro-4,4′-bi­pyrazole [H_2_(TNBP)] was synthesized in 92% yield by nitration of 4,4′-bi­pyrazole in mixed acids and then crystallized from water as a monohydrate (Domasevitch *et al.*, 2019 ▸).

To prepare the Rb salt (**1**), 0.332 g (1.0 mmol) of H_2_(TNBP)·H_2_O was added to a solution of 0.116 g (0.5 mmol) of Rb_2_CO_3_ in 8 ml of water and the mixture was heated at 353–363 K until total dissolution was observed. The solution was cooled to room temperature and left for a few hours for crystallization. Pale-yellow crystals of Rb{H(TNBP)} were isolated in a yield of 0.325 g (82%) and dried in air. The compound is unstable when stored under the reaction solution as the initially formed crystals dissolve in a period of 10–15 d and colorless H_2_(TNBP)·H_2_O deposits. In a similar way, the reaction of 0.332 g (1.0 mmol) of H_2_(TNBP)·H_2_O and 0.163 g (0.5 mmol) of Cs_2_CO_3_ in 8 ml of water gives 0.415 g (93%) of pale-yellow Cs{H(TNBP)} (**2**). Unlike (**1**), this material is stable under the mother solution. Similar reactions with Na_2_CO_3_ and K_2_CO_3_ did not afford any hydrogen bipyrazolates and led to soluble *M*_2_{TNBP} (*M* = Na, K) and precipitation of the excess amount of H_2_(TNBP)·H_2_O.

Analysis (%) calculated for (**1**), C_6_HN_8_O_8_Rb: C 18.08, H 0.25, N 28.12; found: C 17.93, H 0.44, N 28.49. IR (KBr, cm^−1^): 590 *w*, 708 *w*, 838 *m*, 854 *s*, 996 *m*, 1024 *m*, 1308 *s*, 1352 *vs*, 1398 *vs*, 1432 *m*, 1490 *vs*, 1500 *m*, 1556 *vs*, 1636 *w*, 3448 *br*.

Analysis (%) calculated for (**2**), C_6_HCsN_8_O_8_: C 16.15, H 0.23, N 25.13; found: C 16.01, H 0.38, N 28.11. IR (KBr, cm^−1^): 516 *w*, 586 *m*, 708 *m*, 838 *s*, 852 *s*, 994 *s*, 1022 *m*, 1170 *w*, 1306 *s*, 1324 *s*, 1350 *vs*, 1396 *vs*, 1432 *s*, 1488 *vs*, 1512 *vs*, 1544 *vs*, 1634 *m*, 3024 *br*, 3442 *br*.

## Refinement

Crystal data, data collection and structure refinement details are summarized in Table 6 ▸. The hydrogen atoms were located and then refined as riding with N—H = 0.87 Å and *U*_iso_(H) = 1.5*U*_eq_(N). For (**2**), the H atom is equally disordered over two positions corresponding to the N1 and N4 carrier atoms.

## Supplementary Material

Crystal structure: contains datablock(s) global, 1, 2. DOI: 10.1107/S2056989021010227/hb7988sup1.cif

Structure factors: contains datablock(s) 1. DOI: 10.1107/S2056989021010227/hb79881sup2.hkl

Structure factors: contains datablock(s) 2. DOI: 10.1107/S2056989021010227/hb79882sup3.hkl

CCDC references: 2113578, 2113577

Additional supporting information:  crystallographic information; 3D view; checkCIF report

## Figures and Tables

**Table 1:** Selected bond lengths (Å) for (**1**)

Rb1—O1^i^	2.8543 (15)	Rb2—O5^iii^	2.9616 (17)
Rb1—O5	2.9673 (16)	Rb2—O7	2.9690 (15)
Rb1—N3	3.1285 (16)	Rb2—O3^i^	3.0743 (17)
Rb1—O8^ii^	3.3074 (16)	Rb2—N2^i^	3.2261 (16)
Rb1—O3^iii^	3.424 (2)	Rb2—O6^iii^	3.2275 (16)
Rb1—O4^iii^	3.4942 (16)	Rb2—O2^ii^	3.6985 (16)

Symmetry codes: (i) x, y+1, z; (ii) -x+{\script{1\over 2}}, -y+{\script{1\over 2}}, -z; (iii) -x+{\script{1\over 2}}, y+{\script{1\over 2}}, -z+{\script{1\over 2}}.

**Table 2:** Selected bond lengths (Å) for (**2**)

Cs1—O1^i^	3.071 (2)	Cs2—O7	3.109 (2)
Cs1—O5	3.177 (2)	Cs2—O5^ii^	3.159 (2)
Cs1—O3^ii^	3.351 (3)	Cs2—O3^i^	3.297 (2)
Cs1—N3	3.369 (2)	Cs2—O6^ii^	3.396 (2)
Cs1—O4^ii^	3.464 (2)	Cs2—N2^i^	3.401 (2)
Cs1—O8^iii^	3.514 (2)	Cs2—O2^iv^	3.811 (2)

Symmetry codes: (i) x, y+1, z; (ii) -x+{\script{1\over 2}}, y+{\script{1\over 2}}, -z+{\script{1\over 2}}; (iii) -x+{\script{1\over 2}}, -y+{\script{1\over 2}}, -z; (iv) x-{\script{1\over 2}}, y+{\script{1\over 2}}, z.

**Table 3:** Geometry of lone pair–π-hole inter­actions (Å, °) in (**1**) and (**2**) N⋯plane is a distance of an N-donor to the mean plane of a nitro group and φ is an angle of the N⋯N axis to the plane of the nitro group.

Compound	N-Donor	Group	N⋯N	N⋯plane	φ
(**1**)	N2	(C4N7O5O6)^xii^	2.997 (3)	2.980 (2)	83.9 (2)
	N3	(C3N6O3O4)^ii^	3.198 (3)	3.093 (2)	75.3 (2)
(**2**)	N2	(C4N7O5O6)^xii^	2.990 (3)	2.976 (3)	84.5 (2)
	N3	(C3N6O3O4)^ii^	3.186 (3)	3.083 (3)	75.4 (2)

Symmetry codes: (ii) −*x* + {1\over 2}, *y* + {1\over 2}, −*z* + {1\over 2}; (xii) −*x* + {1\over 2}, *y* − {1\over 2}, −*z* + {1\over 2}.

**Table 4:** Geometry of stacking inter­actions involving nitro and pyrazole groups (Å, °) in (**1**) and (**2**) Atom⋯*Cg* is the shortest distance from the nitro group atom to the centroid of the ring; Atom⋯plane is the deviation of the given atom from the mean plane of the ring and φ is the angle of the atom⋯*Cg* axis to the plane of the ring.

Compound	Atom	Ring	Atom⋯*Cg*	Atom⋯plane	φ
(**1**)	O7	(C4C5C6N3N4)^iii^	3.265 (3)	3.262 (2)	87.5 (2)
	N5	(C1C2C3N1N2)^xiii^	3.541 (3)	3.526 (3)	84.7 (2)
(**2**)	O7	(C4C5C6N3N4)^iii^	3.240 (3)	3.232 (3)	86.0 (3)
	N5	(C1C2C3N1N2)^xiii^	3.448 (3)	3.389 (3)	79.4 (3)

Symmetry codes: (iii) −*x* + {1\over 2}, −*y* + {1\over 2}, −*z*; (xiii) −*x* + {1\over 2}, −*y* − {1\over 2}, −*z*.

**Table 5:** Contributions of the different kinds of the contacts (%) to the Hirshfeld surfaces of individual anions in (**1**)^*a*^ and (**2**) *M* = Rb (**1**) and Cs (**2**)

Contact	(**1**)	(**2**)
O⋯*M*	13.0	13.6
N⋯*M*	2.4	2.2
O⋯O	37.4	35.5
N⋯N	5.3	6.3
C⋯C	1.0	0.7
O⋯N/N⋯O	15.8	16.2
O⋯C/C⋯O	3.8	5.4
N⋯C/C⋯N	7.5	6.6
N⋯H/H⋯N	6.9	6.6
O⋯H/H⋯O	5.4	5.2
C⋯H/H⋯C	1.5	1.7

Note: (*a*) For the two-dimensional plots for (**1**), see Fig. 7 ▸.

**Table 6:** Experimental details

	(**1**)	(**2**)
Crystal data		
Chemical formula	[Rb(C_6_HN_8_O_8_)]	[Cs(C_6_HN_8_O_8_)]
*M*_r_	398.62	446.06
Crystal system, space group	Monoclinic, *C*2/*c*	Monoclinic, *C*2/*c*
Temperature (K)	213	213
*a*, *b*, *c* (Å)	19.4400 (15), 8.6070 (4), 16.0977 (10)	19.944 (2), 8.6307 (7), 16.2083 (17)
β (°)	115.264 (7)	113.766 (8)
*V* (Å^3^)	2435.8 (3)	2553.4 (5)
*Z*	8	8
Radiation type	Mo *K*α	Mo *K*α
μ (mm^−1^)	4.13	2.97
Crystal size (mm)	0.20 × 0.16 × 0.14	0.20 × 0.16 × 0.14

Data collection		
Diffractometer	Stoe IPDS	Stoe IPDS
Absorption correction	Numerical [*X-RED* (Stoe & Cie, 2001 ▸) and *X-SHAPE* (Stoe & Cie, 1999 ▸)]	Numerical [*X-RED* (Stoe & Cie, 2001 ▸) and *X-SHAPE* (Stoe & Cie, 1999 ▸)]
*T*_min_, *T*_max_	0.672, 0.789	0.677, 0.772
No. of measured, independent and observed [*I* > 2σ(*I*)] reflections	9925, 2890, 2186	9014, 2990, 2686
*R*_int_	0.033	0.042
(sin θ/λ)_max_ (Å^−1^)	0.658	0.656

Refinement		
*R*[*F* ^2^ > 2σ(*F* ^2^)], *wR*(*F* ^2^), *S*	0.026, 0.055, 0.89	0.027, 0.060, 1.21
No. of reflections	2890	2990
No. of parameters	210	211
H-atom treatment	H-atom parameters constrained	H-atom parameters constrained
Δρ_max_, Δρ_min_ (e Å^−3^)	0.37, −0.39	0.67, −0.77

Computer programs: *IPDS Software* (Stoe & Cie, 2000 ▸), *SHELXS97* (Sheldrick, 2008 ▸), *SHELXL2018/1* (Sheldrick, 2015 ▸), *DIAMOND* (Brandenburg, 1999 ▸) and *WinGX* (Farrugia, 2012 ▸).

## supplementary crystallographic information

### Poly[[µ4-4-(3,5-dinitropyrazol-4-yl)-3,5-dinitropyrazol-1-ido]rubidium] (1) Crystal data

**Table:**

[Rb(C_6_HN_8_O_8_)]	*F*(000) = 1552
*M**_r_* = 398.62	*D*_x_ = 2.174 Mg m^−^^3^
Monoclinic, *C*2/*c*	Mo *K*α radiation, λ = 0.71073 Å
*a* = 19.4400 (15) Å	Cell parameters from 8000 reflections
*b* = 8.6070 (4) Å	θ = 2.3–27.9°
*c* = 16.0977 (10) Å	µ = 4.13 mm^−^^1^
β = 115.264 (7)°	*T* = 213 K
*V* = 2435.8 (3) Å^3^	Prism, yellow
*Z* = 8	0.20 × 0.16 × 0.14 mm

### Poly[[µ4-4-(3,5-dinitropyrazol-4-yl)-3,5-dinitropyrazol-1-ido]rubidium] (1) Data collection

**Table:**

Stoe IPDS diffractometer	2186 reflections with *I* > 2σ(*I*)
Radiation source: fine-focus sealed tube	*R*_int_ = 0.033
φ oscillation scans	θ_max_ = 27.9°, θ_min_ = 2.3°
Absorption correction: numerical [X-RED (Stoe & Cie, 2001) and X-SHAPE (Stoe & Cie, 1999)]	*h* = −25→25
*T*_min_ = 0.672, *T*_max_ = 0.789	*k* = −10→11
9925 measured reflections	*l* = −20→20
2890 independent reflections	

### Poly[[µ4-4-(3,5-dinitropyrazol-4-yl)-3,5-dinitropyrazol-1-ido]rubidium] (1) Refinement

**Table:**

Refinement on *F*^2^	Primary atom site location: structure-invariant direct methods
Least-squares matrix: full	Secondary atom site location: difference Fourier map
*R*[*F*^2^ > 2σ(*F*^2^)] = 0.026	Hydrogen site location: inferred from neighbouring sites
*wR*(*F*^2^) = 0.055	H-atom parameters constrained
*S* = 0.89	*w* = 1/[σ^2^(*F*_o_^2^) + (0.0326*P*)^2^] where *P* = (*F*_o_^2^ + 2*F*_c_^2^)/3
2890 reflections	(Δ/σ)_max_ < 0.001
210 parameters	Δρ_max_ = 0.37 e Å^−^^3^
0 restraints	Δρ_min_ = −0.39 e Å^−^^3^

### Poly[[µ4-4-(3,5-dinitropyrazol-4-yl)-3,5-dinitropyrazol-1-ido]rubidium] (1) Special details

**Table:**

Geometry. All esds (except the esd in the dihedral angle between two l.s. planes) are estimated using the full covariance matrix. The cell esds are taken into account individually in the estimation of esds in distances, angles and torsion angles; correlations between esds in cell parameters are only used when they are defined by crystal symmetry. An approximate (isotropic) treatment of cell esds is used for estimating esds involving l.s. planes.

### Poly[[µ4-4-(3,5-dinitropyrazol-4-yl)-3,5-dinitropyrazol-1-ido]rubidium] (1) Fractional atomic coordinates and isotropic or equivalent isotropic displacement parameters (Å^2^)

**Table:**

	*x*	*y*	*z*	*U*_iso_*/*U*_eq_	
Rb1	0.500000	0.39279 (3)	0.250000	0.02840 (9)	
Rb2	0.000000	0.500000	0.000000	0.03230 (9)	
O1	0.38330 (9)	−0.38304 (18)	0.16106 (14)	0.0422 (5)	
O2	0.38489 (9)	−0.14611 (18)	0.11639 (13)	0.0344 (4)	
O3	0.03697 (9)	−0.16032 (19)	0.06215 (16)	0.0490 (5)	
O4	0.10681 (9)	0.03244 (17)	0.13792 (13)	0.0347 (4)	
O5	0.45319 (8)	0.09854 (18)	0.30718 (12)	0.0329 (4)	
O6	0.37797 (9)	−0.09294 (16)	0.29580 (11)	0.0301 (4)	
O7	0.13123 (9)	0.34370 (17)	−0.01293 (13)	0.0344 (4)	
O8	0.12218 (8)	0.09417 (17)	−0.03095 (11)	0.0280 (3)	
N1	0.23222 (9)	−0.36612 (17)	0.10737 (13)	0.0199 (4)	
H1	0.247444	−0.461154	0.107308	0.030*	
N2	0.16218 (9)	−0.32601 (19)	0.09532 (13)	0.0210 (4)	
N3	0.33470 (9)	0.27100 (18)	0.19547 (13)	0.0207 (4)	
N4	0.26946 (9)	0.31935 (17)	0.12643 (13)	0.0200 (4)	
N5	0.35387 (9)	−0.25662 (18)	0.13349 (12)	0.0212 (4)	
N6	0.09790 (10)	−0.0935 (2)	0.10011 (14)	0.0266 (4)	
N7	0.39083 (9)	0.03467 (19)	0.27152 (13)	0.0218 (4)	
N8	0.15509 (9)	0.21089 (19)	0.01062 (13)	0.0206 (4)	
C1	0.27606 (10)	−0.2391 (2)	0.11965 (14)	0.0175 (4)	
C2	0.23545 (10)	−0.1061 (2)	0.11814 (14)	0.0161 (4)	
C3	0.16475 (10)	−0.1719 (2)	0.10251 (15)	0.0181 (4)	
C4	0.32927 (10)	0.1159 (2)	0.19966 (14)	0.0177 (4)	
C5	0.26131 (10)	0.0553 (2)	0.13416 (14)	0.0158 (4)	
C6	0.22651 (10)	0.1926 (2)	0.08941 (14)	0.0167 (4)	

### Poly[[µ4-4-(3,5-dinitropyrazol-4-yl)-3,5-dinitropyrazol-1-ido]rubidium] (1) Atomic displacement parameters (Å^2^)

**Table:**

	*U*^11^	*U*^22^	*U*^33^	*U*^12^	*U*^13^	*U*^23^
Rb1	0.01707 (13)	0.01962 (14)	0.0442 (2)	0.000	0.00897 (13)	0.000
Rb2	0.02226 (15)	0.03355 (17)	0.0307 (2)	0.00090 (12)	0.00132 (12)	−0.01165 (14)
O1	0.0311 (8)	0.0188 (8)	0.0702 (14)	0.0128 (6)	0.0153 (9)	0.0038 (8)
O2	0.0284 (8)	0.0279 (8)	0.0533 (12)	−0.0005 (6)	0.0234 (8)	0.0034 (8)
O3	0.0212 (8)	0.0283 (9)	0.0941 (17)	−0.0011 (7)	0.0214 (9)	0.0036 (9)
O4	0.0411 (9)	0.0214 (8)	0.0519 (12)	0.0071 (7)	0.0299 (9)	−0.0003 (7)
O5	0.0206 (7)	0.0336 (8)	0.0327 (10)	−0.0044 (6)	0.0001 (7)	0.0048 (7)
O6	0.0375 (8)	0.0155 (7)	0.0272 (10)	−0.0001 (6)	0.0040 (7)	0.0046 (6)
O7	0.0313 (8)	0.0190 (7)	0.0470 (12)	0.0121 (6)	0.0111 (8)	0.0127 (7)
O8	0.0225 (7)	0.0231 (8)	0.0305 (10)	−0.0046 (6)	0.0037 (6)	0.0008 (7)
N1	0.0231 (8)	0.0089 (7)	0.0267 (11)	0.0004 (6)	0.0098 (7)	−0.0010 (7)
N2	0.0222 (8)	0.0127 (8)	0.0283 (11)	−0.0004 (6)	0.0109 (7)	0.0020 (7)
N3	0.0220 (8)	0.0141 (8)	0.0230 (10)	−0.0016 (6)	0.0067 (7)	−0.0010 (7)
N4	0.0213 (8)	0.0103 (7)	0.0277 (11)	−0.0001 (6)	0.0098 (7)	0.0009 (7)
N5	0.0211 (8)	0.0164 (8)	0.0230 (10)	0.0031 (6)	0.0064 (7)	−0.0051 (7)
N6	0.0256 (9)	0.0174 (9)	0.0423 (13)	0.0030 (7)	0.0198 (8)	0.0077 (8)
N7	0.0241 (9)	0.0176 (9)	0.0196 (10)	0.0010 (6)	0.0053 (7)	−0.0014 (7)
N8	0.0187 (8)	0.0180 (8)	0.0263 (11)	0.0031 (6)	0.0108 (7)	0.0057 (7)
C1	0.0203 (9)	0.0115 (9)	0.0193 (12)	0.0001 (7)	0.0070 (8)	0.0000 (7)
C2	0.0187 (9)	0.0104 (8)	0.0188 (11)	0.0010 (7)	0.0075 (8)	0.0008 (8)
C3	0.0203 (9)	0.0122 (9)	0.0223 (12)	0.0008 (7)	0.0095 (8)	0.0019 (8)
C4	0.0206 (9)	0.0119 (8)	0.0192 (11)	0.0007 (7)	0.0070 (8)	−0.0003 (7)
C5	0.0177 (9)	0.0109 (8)	0.0202 (12)	0.0014 (7)	0.0094 (8)	0.0003 (7)
C6	0.0185 (9)	0.0126 (8)	0.0198 (11)	0.0016 (7)	0.0089 (8)	0.0011 (7)

### Poly[[µ4-4-(3,5-dinitropyrazol-4-yl)-3,5-dinitropyrazol-1-ido]rubidium] (1) Geometric parameters (Å, º)

**Table:**

Rb1—O1^i^	2.8543 (15)	O1—N5	1.221 (2)
Rb1—O1^ii^	2.8543 (15)	O2—N5	1.219 (2)
Rb1—O5	2.9673 (16)	O3—N6	1.220 (2)
Rb1—O5^iii^	2.9673 (16)	O4—N6	1.219 (2)
Rb1—N3	3.1285 (16)	O5—N7	1.228 (2)
Rb1—N3^iii^	3.1285 (16)	O6—N7	1.227 (2)
Rb1—O8^iv^	3.3074 (16)	O7—N8	1.231 (2)
Rb1—O8^v^	3.3074 (16)	O8—N8	1.225 (2)
Rb1—O3^vi^	3.424 (2)	N1—N2	1.336 (2)
Rb1—O3^vii^	3.424 (2)	N1—C1	1.348 (2)
Rb1—O4^vi^	3.4942 (16)	N1—H1	0.8700
Rb1—O4^vii^	3.4942 (16)	N2—C3	1.331 (2)
Rb2—O5^viii^	2.9616 (17)	N3—C4	1.343 (2)
Rb2—O5^vii^	2.9616 (17)	N3—N4	1.347 (2)
Rb2—O7^ix^	2.9690 (15)	N4—C6	1.348 (2)
Rb2—O7	2.9690 (15)	N5—C1	1.440 (2)
Rb2—O3^x^	3.0743 (17)	N6—C3	1.450 (2)
Rb2—O3^ii^	3.0743 (17)	N7—C4	1.441 (2)
Rb2—N2^x^	3.2261 (16)	N8—C6	1.435 (3)
Rb2—N2^ii^	3.2261 (16)	C1—C2	1.384 (3)
Rb2—O6^viii^	3.2275 (16)	C2—C3	1.406 (3)
Rb2—O6^vii^	3.2275 (16)	C2—C5	1.462 (2)
Rb2—O2^v^	3.6985 (16)	C4—C5	1.393 (3)
Rb2—O2^xi^	3.6985 (16)	C5—C6	1.399 (3)
			
O1^i^—Rb1—O1^ii^	94.94 (7)	O5^vii^—Rb2—O6^viii^	139.46 (4)
O1^i^—Rb1—O5	134.80 (5)	O7^ix^—Rb2—O6^viii^	71.28 (5)
O1^ii^—Rb1—O5	116.69 (4)	O7—Rb2—O6^viii^	108.72 (5)
O1^i^—Rb1—O5^iii^	116.69 (4)	O3^x^—Rb2—O6^viii^	86.34 (5)
O1^ii^—Rb1—O5^iii^	134.80 (5)	O3^ii^—Rb2—O6^viii^	93.66 (5)
O5—Rb1—O5^iii^	62.80 (7)	N2^x^—Rb2—O6^viii^	59.09 (4)
O1^i^—Rb1—N3	150.29 (5)	N2^ii^—Rb2—O6^viii^	120.91 (4)
O1^ii^—Rb1—N3	65.44 (5)	O5^viii^—Rb2—O6^vii^	139.46 (4)
O5—Rb1—N3	52.18 (4)	O5^vii^—Rb2—O6^vii^	40.54 (4)
O5^iii^—Rb1—N3	92.36 (4)	O7^ix^—Rb2—O6^vii^	108.72 (5)
O1^i^—Rb1—N3^iii^	65.44 (5)	O7—Rb2—O6^vii^	71.28 (5)
O1^ii^—Rb1—N3^iii^	150.29 (5)	O3^x^—Rb2—O6^vii^	93.66 (5)
O5—Rb1—N3^iii^	92.36 (4)	O3^ii^—Rb2—O6^vii^	86.34 (5)
O5^iii^—Rb1—N3^iii^	52.18 (4)	N2^x^—Rb2—O6^vii^	120.91 (4)
N3—Rb1—N3^iii^	140.85 (6)	N2^ii^—Rb2—O6^vii^	59.09 (4)
O1^i^—Rb1—O8^iv^	52.19 (5)	O6^viii^—Rb2—O6^vii^	180.00 (7)
O1^ii^—Rb1—O8^iv^	124.56 (5)	O5^viii^—Rb2—O2^v^	63.14 (4)
O5—Rb1—O8^iv^	82.76 (4)	O5^vii^—Rb2—O2^v^	116.86 (4)
O5^iii^—Rb1—O8^iv^	100.60 (4)	O7^ix^—Rb2—O2^v^	127.24 (4)
N3—Rb1—O8^iv^	119.65 (4)	O7—Rb2—O2^v^	52.75 (4)
N3^iii^—Rb1—O8^iv^	61.84 (4)	O3^x^—Rb2—O2^v^	105.45 (5)
O1^i^—Rb1—O8^v^	124.56 (5)	O3^ii^—Rb2—O2^v^	74.55 (5)
O1^ii^—Rb1—O8^v^	52.19 (5)	N2^x^—Rb2—O2^v^	126.63 (4)
O5—Rb1—O8^v^	100.60 (4)	N2^ii^—Rb2—O2^v^	53.37 (4)
O5^iii^—Rb1—O8^v^	82.76 (4)	O6^viii^—Rb2—O2^v^	74.96 (4)
N3—Rb1—O8^v^	61.84 (4)	O6^vii^—Rb2—O2^v^	105.04 (4)
N3^iii^—Rb1—O8^v^	119.65 (4)	O5^viii^—Rb2—O2^xi^	116.86 (4)
O8^iv^—Rb1—O8^v^	176.11 (5)	O5^vii^—Rb2—O2^xi^	63.14 (4)
O1^i^—Rb1—O3^vi^	96.42 (5)	O7^ix^—Rb2—O2^xi^	52.76 (4)
O1^ii^—Rb1—O3^vi^	93.94 (5)	O7—Rb2—O2^xi^	127.25 (4)
O5—Rb1—O3^vi^	111.58 (4)	O3^x^—Rb2—O2^xi^	74.55 (5)
O5^iii^—Rb1—O3^vi^	53.44 (4)	O3^ii^—Rb2—O2^xi^	105.45 (5)
N3—Rb1—O3^vi^	106.56 (4)	N2^x^—Rb2—O2^xi^	53.37 (4)
N3^iii^—Rb1—O3^vi^	68.00 (4)	N2^ii^—Rb2—O2^xi^	126.63 (4)
O8^iv^—Rb1—O3^vi^	128.33 (4)	O6^viii^—Rb2—O2^xi^	105.04 (4)
O8^v^—Rb1—O3^vi^	52.33 (4)	O6^vii^—Rb2—O2^xi^	74.96 (4)
O1^i^—Rb1—O3^vii^	93.94 (5)	O2^v^—Rb2—O2^xi^	180.00 (3)
O1^ii^—Rb1—O3^vii^	96.42 (5)	N5—O1—Rb1^xii^	158.96 (13)
O5—Rb1—O3^vii^	53.44 (4)	N6—O3—Rb2^xii^	130.77 (13)
O5^iii^—Rb1—O3^vii^	111.58 (4)	N6—O3—Rb1^xiii^	91.12 (15)
N3—Rb1—O3^vii^	68.00 (4)	Rb2^xii^—O3—Rb1^xiii^	107.84 (5)
N3^iii^—Rb1—O3^vii^	106.56 (4)	N6—O4—Rb1^xiii^	87.86 (12)
O8^iv^—Rb1—O3^vii^	52.33 (4)	N7—O5—Rb2^xiv^	99.33 (12)
O8^v^—Rb1—O3^vii^	128.33 (4)	N7—O5—Rb1	127.55 (12)
O3^vi^—Rb1—O3^vii^	164.66 (6)	Rb2^xiv^—O5—Rb1	124.88 (5)
O1^i^—Rb1—O4^vi^	60.41 (5)	N7—O6—Rb2^xiv^	86.64 (11)
O1^ii^—Rb1—O4^vi^	91.65 (5)	N8—O7—Rb2	128.40 (13)
O5—Rb1—O4^vi^	141.47 (4)	N8—O8—Rb1^v^	121.64 (12)
O5^iii^—Rb1—O4^vi^	78.75 (4)	N2—N1—C1	110.67 (15)
N3—Rb1—O4^vi^	137.39 (5)	N2—N1—H1	124.7
N3^iii^—Rb1—O4^vi^	59.64 (4)	C1—N1—H1	124.7
O8^iv^—Rb1—O4^vi^	102.96 (4)	C3—N2—N1	104.29 (15)
O8^v^—Rb1—O4^vi^	75.66 (4)	C3—N2—Rb2^xii^	119.80 (12)
O3^vi^—Rb1—O4^vi^	36.38 (4)	N1—N2—Rb2^xii^	132.66 (12)
O3^vii^—Rb1—O4^vi^	153.75 (4)	C4—N3—N4	106.38 (15)
O1^i^—Rb1—O4^vii^	91.65 (5)	C4—N3—Rb1	114.35 (12)
O1^ii^—Rb1—O4^vii^	60.41 (5)	N4—N3—Rb1	128.37 (12)
O5—Rb1—O4^vii^	78.75 (4)	N3—N4—C6	107.59 (15)
O5^iii^—Rb1—O4^vii^	141.47 (4)	O2—N5—O1	125.25 (17)
N3—Rb1—O4^vii^	59.64 (4)	O2—N5—C1	118.13 (15)
N3^iii^—Rb1—O4^vii^	137.39 (5)	O1—N5—C1	116.61 (16)
O8^iv^—Rb1—O4^vii^	75.66 (4)	O4—N6—O3	124.73 (18)
O8^v^—Rb1—O4^vii^	102.96 (4)	O4—N6—C3	117.74 (17)
O3^vi^—Rb1—O4^vii^	153.75 (4)	O3—N6—C3	117.50 (18)
O3^vii^—Rb1—O4^vii^	36.38 (4)	O4—N6—Rb1^xiii^	72.68 (11)
O4^vi^—Rb1—O4^vii^	139.76 (5)	O3—N6—Rb1^xiii^	69.40 (14)
O5^viii^—Rb2—O5^vii^	180.00 (8)	C3—N6—Rb1^xiii^	132.87 (13)
O5^viii^—Rb2—O7^ix^	108.32 (4)	O6—N7—O5	123.15 (18)
O5^vii^—Rb2—O7^ix^	71.68 (4)	O6—N7—C4	118.54 (16)
O5^viii^—Rb2—O7	71.68 (4)	O5—N7—C4	118.29 (16)
O5^vii^—Rb2—O7	108.32 (4)	O6—N7—Rb2^xiv^	72.15 (11)
O7^ix^—Rb2—O7	180.0	O5—N7—Rb2^xiv^	59.69 (11)
O5^viii^—Rb2—O3^x^	57.45 (5)	C4—N7—Rb2^xiv^	146.67 (12)
O5^vii^—Rb2—O3^x^	122.55 (5)	O8—N8—O7	123.57 (18)
O7^ix^—Rb2—O3^x^	111.42 (4)	O8—N8—C6	118.40 (15)
O7—Rb2—O3^x^	68.58 (4)	O7—N8—C6	118.00 (17)
O5^viii^—Rb2—O3^ii^	122.55 (5)	N1—C1—C2	110.36 (16)
O5^vii^—Rb2—O3^ii^	57.45 (5)	N1—C1—N5	119.62 (15)
O7^ix^—Rb2—O3^ii^	68.58 (4)	C2—C1—N5	130.01 (16)
O7—Rb2—O3^ii^	111.42 (4)	C1—C2—C3	100.20 (16)
O3^x^—Rb2—O3^ii^	180.00 (10)	C1—C2—C5	129.17 (17)
O5^viii^—Rb2—N2^x^	64.39 (4)	C3—C2—C5	130.53 (17)
O5^vii^—Rb2—N2^x^	115.61 (4)	N2—C3—C2	114.47 (16)
O7^ix^—Rb2—N2^x^	63.20 (4)	N2—C3—N6	117.47 (16)
O7—Rb2—N2^x^	116.80 (4)	C2—C3—N6	127.90 (17)
O3^x^—Rb2—N2^x^	50.09 (4)	N3—C4—C5	113.82 (17)
O3^ii^—Rb2—N2^x^	129.91 (4)	N3—C4—N7	117.80 (17)
O5^viii^—Rb2—N2^ii^	115.61 (4)	C5—C4—N7	128.31 (17)
O5^vii^—Rb2—N2^ii^	64.39 (4)	C4—C5—C6	99.63 (15)
O7^ix^—Rb2—N2^ii^	116.80 (4)	C4—C5—C2	129.27 (17)
O7—Rb2—N2^ii^	63.20 (4)	C6—C5—C2	131.10 (17)
O3^x^—Rb2—N2^ii^	129.91 (4)	N4—C6—C5	112.57 (17)
O3^ii^—Rb2—N2^ii^	50.09 (4)	N4—C6—N8	119.01 (16)
N2^x^—Rb2—N2^ii^	180.0	C5—C6—N8	128.38 (16)
O5^viii^—Rb2—O6^viii^	40.54 (4)		
			
C1—N1—N2—C3	−1.3 (2)	C1—C2—C3—N2	0.0 (2)
C1—N1—N2—Rb2^xii^	157.43 (14)	C5—C2—C3—N2	−176.6 (2)
C4—N3—N4—C6	−1.0 (2)	C1—C2—C3—N6	175.2 (2)
Rb1—N3—N4—C6	140.39 (14)	C5—C2—C3—N6	−1.4 (4)
Rb1^xii^—O1—N5—O2	26.7 (6)	O4—N6—C3—N2	154.3 (2)
Rb1^xii^—O1—N5—C1	−153.8 (4)	O3—N6—C3—N2	−24.1 (3)
Rb1^xiii^—O4—N6—O3	48.3 (2)	Rb1^xiii^—N6—C3—N2	62.6 (3)
Rb1^xiii^—O4—N6—C3	−129.88 (17)	O4—N6—C3—C2	−20.8 (3)
Rb2^xii^—O3—N6—O4	−164.56 (17)	O3—N6—C3—C2	160.8 (2)
Rb1^xiii^—O3—N6—O4	−49.6 (2)	Rb1^xiii^—N6—C3—C2	−112.5 (2)
Rb2^xii^—O3—N6—C3	13.7 (3)	N4—N3—C4—C5	0.3 (2)
Rb1^xiii^—O3—N6—C3	128.59 (17)	Rb1—N3—C4—C5	−147.15 (14)
Rb2^xii^—O3—N6—Rb1^xiii^	−114.92 (19)	N4—N3—C4—N7	−177.06 (17)
Rb2^xiv^—O6—N7—O5	32.5 (2)	Rb1—N3—C4—N7	35.5 (2)
Rb2^xiv^—O6—N7—C4	−145.49 (16)	O6—N7—C4—N3	156.77 (19)
Rb2^xiv^—O5—N7—O6	−36.3 (2)	O5—N7—C4—N3	−21.3 (3)
Rb1—O5—N7—O6	174.67 (14)	Rb2^xiv^—N7—C4—N3	55.7 (3)
Rb2^xiv^—O5—N7—C4	141.67 (15)	O6—N7—C4—C5	−20.2 (3)
Rb1—O5—N7—C4	−7.3 (3)	O5—N7—C4—C5	161.7 (2)
Rb1—O5—N7—Rb2^xiv^	−149.01 (17)	Rb2^xiv^—N7—C4—C5	−121.3 (2)
Rb1^v^—O8—N8—O7	22.7 (3)	N3—C4—C5—C6	0.4 (2)
Rb1^v^—O8—N8—C6	−155.32 (13)	N7—C4—C5—C6	177.5 (2)
Rb2—O7—N8—O8	72.4 (3)	N3—C4—C5—C2	−179.85 (19)
Rb2—O7—N8—C6	−109.63 (17)	N7—C4—C5—C2	−2.8 (4)
N2—N1—C1—C2	1.4 (2)	C1—C2—C5—C4	−41.0 (4)
N2—N1—C1—N5	−179.72 (17)	C3—C2—C5—C4	134.7 (2)
O2—N5—C1—N1	157.8 (2)	C1—C2—C5—C6	138.7 (2)
O1—N5—C1—N1	−21.7 (3)	C3—C2—C5—C6	−45.6 (4)
O2—N5—C1—C2	−23.6 (3)	N3—N4—C6—C5	1.3 (2)
O1—N5—C1—C2	156.9 (2)	N3—N4—C6—N8	−176.64 (16)
N1—C1—C2—C3	−0.8 (2)	C4—C5—C6—N4	−1.0 (2)
N5—C1—C2—C3	−179.5 (2)	C2—C5—C6—N4	179.2 (2)
N1—C1—C2—C5	175.8 (2)	C4—C5—C6—N8	176.7 (2)
N5—C1—C2—C5	−2.9 (4)	C2—C5—C6—N8	−3.1 (4)
N1—N2—C3—C2	0.8 (2)	O8—N8—C6—N4	168.87 (18)
Rb2^xii^—N2—C3—C2	−161.31 (14)	O7—N8—C6—N4	−9.2 (3)
N1—N2—C3—N6	−174.94 (18)	O8—N8—C6—C5	−8.7 (3)
Rb2^xii^—N2—C3—N6	23.0 (2)	O7—N8—C6—C5	173.2 (2)

Symmetry codes: (i) −*x*+1, *y*+1, −*z*+1/2; (ii) *x*, *y*+1, *z*; (iii) −*x*+1, *y*, −*z*+1/2; (iv) *x*+1/2, −*y*+1/2, *z*+1/2; (v) −*x*+1/2, −*y*+1/2, −*z*; (vi) *x*+1/2, *y*+1/2, *z*; (vii) −*x*+1/2, *y*+1/2, −*z*+1/2; (viii) *x*−1/2, −*y*+1/2, *z*−1/2; (ix) −*x*, −*y*+1, −*z*; (x) −*x*, −*y*, −*z*; (xi) *x*−1/2, *y*+1/2, *z*; (xii) *x*, *y*−1, *z*; (xiii) *x*−1/2, *y*−1/2, *z*; (xiv) −*x*+1/2, *y*−1/2, −*z*+1/2.

### Poly[[µ4-4-(3,5-dinitropyrazol-4-yl)-3,5-dinitropyrazol-1-ido]rubidium] (1) Hydrogen-bond geometry (Å, º)

**Table:**

*D*—H···*A*	*D*—H	H···*A*	*D*···*A*	*D*—H···*A*
N1—H1···N4^xii^	0.87	1.93	2.785 (2)	166

Symmetry code: (xii) *x*, *y*−1, *z*.

### Poly[[µ4-4-(3,5-dinitropyrazol-4-yl)-3,5-dinitropyrazol-1-ido]caesium] (2) Crystal data

**Table:**

[Cs(C_6_HN_8_O_8_)]	*F*(000) = 1696
*M**_r_* = 446.06	*D*_x_ = 2.321 Mg m^−^^3^
Monoclinic, *C*2/*c*	Mo *K*α radiation, λ = 0.71073 Å
*a* = 19.944 (2) Å	Cell parameters from 8000 reflections
*b* = 8.6307 (7) Å	θ = 2.2–27.8°
*c* = 16.2083 (17) Å	µ = 2.97 mm^−^^1^
β = 113.766 (8)°	*T* = 213 K
*V* = 2553.4 (5) Å^3^	Prism, yellow
*Z* = 8	0.20 × 0.16 × 0.14 mm

### Poly[[µ4-4-(3,5-dinitropyrazol-4-yl)-3,5-dinitropyrazol-1-ido]caesium] (2) Data collection

**Table:**

Stoe IPDS diffractometer	2686 reflections with *I* > 2σ(*I*)
Radiation source: fine-focus sealed tube	*R*_int_ = 0.042
φ oscillation scans	θ_max_ = 27.8°, θ_min_ = 2.2°
Absorption correction: numerical [X-RED (Stoe & Cie, 2001) and X-SHAPE (Stoe & Cie, 1999)]	*h* = −20→26
*T*_min_ = 0.677, *T*_max_ = 0.772	*k* = −11→11
9014 measured reflections	*l* = −21→21
2990 independent reflections	

### Poly[[µ4-4-(3,5-dinitropyrazol-4-yl)-3,5-dinitropyrazol-1-ido]caesium] (2) Refinement

**Table:**

Refinement on *F*^2^	Secondary atom site location: difference Fourier map
Least-squares matrix: full	Hydrogen site location: inferred from neighbouring sites
*R*[*F*^2^ > 2σ(*F*^2^)] = 0.027	H-atom parameters constrained
*wR*(*F*^2^) = 0.060	*w* = 1/[σ^2^(*F*_o_^2^) + (0.0214*P*)^2^ + 3.932*P*] where *P* = (*F*_o_^2^ + 2*F*_c_^2^)/3
*S* = 1.21	(Δ/σ)_max_ < 0.001
2990 reflections	Δρ_max_ = 0.67 e Å^−^^3^
211 parameters	Δρ_min_ = −0.77 e Å^−^^3^
0 restraints	Extinction correction: *SHELXL2018/1* (Sheldrick 2015), Fc^*^=kFc[1+0.001xFc^2^λ^3^/sin(2θ)]^-1/4^
Primary atom site location: structure-invariant direct methods	Extinction coefficient: 0.00151 (14)

### Poly[[µ4-4-(3,5-dinitropyrazol-4-yl)-3,5-dinitropyrazol-1-ido]caesium] (2) Special details

**Table:**

Geometry. All esds (except the esd in the dihedral angle between two l.s. planes) are estimated using the full covariance matrix. The cell esds are taken into account individually in the estimation of esds in distances, angles and torsion angles; correlations between esds in cell parameters are only used when they are defined by crystal symmetry. An approximate (isotropic) treatment of cell esds is used for estimating esds involving l.s. planes.

### Poly[[µ4-4-(3,5-dinitropyrazol-4-yl)-3,5-dinitropyrazol-1-ido]caesium] (2) Fractional atomic coordinates and isotropic or equivalent isotropic displacement parameters (Å^2^)

**Table:**

	*x*	*y*	*z*	*U*_iso_*/*U*_eq_	Occ. (<1)
Cs1	0.500000	0.42703 (3)	0.250000	0.03031 (9)	
Cs2	0.000000	0.500000	0.000000	0.04080 (10)	
O1	0.36906 (14)	−0.3754 (3)	0.1294 (2)	0.0536 (7)	
O2	0.38087 (12)	−0.1274 (2)	0.12176 (15)	0.0369 (5)	
O3	0.04269 (12)	−0.1420 (3)	0.07349 (18)	0.0460 (6)	
O4	0.11168 (14)	0.0488 (2)	0.14521 (17)	0.0391 (5)	
O5	0.44367 (12)	0.1085 (2)	0.29706 (15)	0.0379 (5)	
O6	0.36857 (13)	−0.0765 (2)	0.29226 (14)	0.0351 (5)	
O7	0.13940 (12)	0.3610 (2)	−0.01257 (15)	0.0391 (5)	
O8	0.12142 (13)	0.1123 (2)	−0.02282 (14)	0.0370 (5)	
N1	0.22944 (13)	−0.3511 (2)	0.10259 (15)	0.0260 (5)	
H1A	0.243307	−0.445633	0.099377	0.039*	0.5
N2	0.16260 (13)	−0.3095 (2)	0.09520 (15)	0.0267 (5)	
N3	0.32972 (13)	0.2858 (2)	0.19322 (15)	0.0266 (5)	
N4	0.26741 (13)	0.3309 (2)	0.12597 (15)	0.0252 (5)	
H1B	0.256042	0.426103	0.108253	0.038*	0.5
N5	0.34581 (13)	−0.2427 (2)	0.12310 (15)	0.0260 (5)	
N6	0.10201 (14)	−0.0772 (3)	0.10736 (17)	0.0304 (5)	
N7	0.38266 (14)	0.0482 (3)	0.26693 (15)	0.0268 (5)	
N8	0.15723 (13)	0.2273 (2)	0.01333 (15)	0.0253 (5)	
C1	0.27188 (15)	−0.2249 (3)	0.11572 (17)	0.0228 (5)	
C2	0.23398 (15)	−0.0903 (3)	0.11868 (16)	0.0216 (5)	
C3	0.16581 (15)	−0.1553 (3)	0.10518 (17)	0.0236 (5)	
C4	0.32386 (15)	0.1316 (3)	0.19768 (17)	0.0230 (5)	
C5	0.25873 (14)	0.0703 (3)	0.13417 (16)	0.0208 (5)	
C6	0.22504 (15)	0.2064 (3)	0.08996 (17)	0.0230 (5)	

### Poly[[µ4-4-(3,5-dinitropyrazol-4-yl)-3,5-dinitropyrazol-1-ido]caesium] (2) Atomic displacement parameters (Å^2^)

**Table:**

	*U*^11^	*U*^22^	*U*^33^	*U*^12^	*U*^13^	*U*^23^
Cs1	0.02186 (13)	0.02958 (13)	0.03945 (15)	0.000	0.01232 (10)	0.000
Cs2	0.02484 (14)	0.04935 (19)	0.03776 (16)	−0.00107 (11)	0.00175 (11)	−0.01535 (12)
O1	0.0407 (13)	0.0213 (10)	0.095 (2)	0.0120 (10)	0.0230 (14)	−0.0019 (12)
O2	0.0366 (12)	0.0257 (10)	0.0524 (13)	0.0006 (9)	0.0220 (10)	0.0023 (9)
O3	0.0277 (11)	0.0353 (11)	0.0727 (16)	−0.0002 (10)	0.0177 (11)	0.0033 (11)
O4	0.0456 (13)	0.0234 (10)	0.0604 (14)	0.0040 (9)	0.0339 (12)	−0.0020 (9)
O5	0.0276 (11)	0.0354 (11)	0.0418 (12)	−0.0014 (8)	0.0047 (9)	0.0046 (9)
O6	0.0440 (13)	0.0198 (9)	0.0305 (10)	−0.0020 (8)	0.0035 (9)	0.0055 (8)
O7	0.0395 (12)	0.0224 (9)	0.0488 (12)	0.0106 (9)	0.0107 (10)	0.0125 (9)
O8	0.0369 (12)	0.0276 (10)	0.0369 (11)	−0.0045 (9)	0.0048 (9)	0.0035 (8)
N1	0.0305 (11)	0.0154 (9)	0.0310 (11)	0.0029 (9)	0.0113 (9)	0.0008 (9)
N2	0.0312 (12)	0.0172 (10)	0.0313 (11)	−0.0004 (9)	0.0121 (10)	0.0011 (9)
N3	0.0305 (12)	0.0187 (10)	0.0282 (11)	−0.0016 (9)	0.0095 (9)	−0.0018 (9)
N4	0.0311 (12)	0.0127 (9)	0.0306 (11)	0.0031 (8)	0.0111 (9)	0.0014 (8)
N5	0.0272 (12)	0.0207 (10)	0.0283 (11)	0.0053 (9)	0.0094 (9)	−0.0017 (8)
N6	0.0314 (13)	0.0229 (11)	0.0427 (14)	0.0025 (10)	0.0208 (11)	0.0070 (10)
N7	0.0299 (12)	0.0211 (10)	0.0254 (11)	0.0028 (9)	0.0069 (9)	−0.0004 (8)
N8	0.0268 (12)	0.0206 (10)	0.0296 (11)	0.0045 (9)	0.0123 (9)	0.0048 (9)
C1	0.0282 (13)	0.0143 (10)	0.0232 (12)	0.0035 (9)	0.0076 (10)	0.0018 (9)
C2	0.0288 (13)	0.0131 (10)	0.0214 (11)	0.0013 (10)	0.0085 (10)	0.0011 (8)
C3	0.0284 (13)	0.0163 (11)	0.0279 (12)	0.0015 (10)	0.0134 (11)	0.0023 (9)
C4	0.0274 (13)	0.0171 (11)	0.0227 (11)	0.0022 (10)	0.0082 (10)	0.0010 (9)
C5	0.0267 (13)	0.0130 (10)	0.0232 (11)	0.0020 (9)	0.0105 (10)	0.0021 (9)
C6	0.0265 (13)	0.0162 (11)	0.0260 (12)	0.0052 (9)	0.0101 (10)	0.0026 (9)

### Poly[[µ4-4-(3,5-dinitropyrazol-4-yl)-3,5-dinitropyrazol-1-ido]caesium] (2) Geometric parameters (Å, º)

**Table:**

Cs1—O1^i^	3.071 (2)	O2—N5	1.222 (3)
Cs1—O1^ii^	3.071 (2)	O3—N6	1.221 (3)
Cs1—O5^iii^	3.177 (2)	O4—N6	1.225 (3)
Cs1—O5	3.177 (2)	O5—N7	1.229 (3)
Cs1—O3^iv^	3.351 (3)	O6—N7	1.224 (3)
Cs1—O3^v^	3.351 (3)	O7—N8	1.230 (3)
Cs1—N3	3.369 (2)	O8—N8	1.225 (3)
Cs1—N3^iii^	3.369 (2)	N1—N2	1.338 (3)
Cs1—O4^iv^	3.464 (2)	N1—C1	1.342 (3)
Cs1—O4^v^	3.464 (2)	N1—H1A	0.8700
Cs1—O8^vi^	3.514 (2)	N2—C3	1.340 (3)
Cs1—O8^vii^	3.514 (2)	N3—N4	1.339 (3)
Cs2—O7^viii^	3.109 (2)	N3—C4	1.340 (3)
Cs2—O7	3.109 (2)	N4—C6	1.346 (3)
Cs2—O5^ix^	3.159 (2)	N4—H1B	0.8700
Cs2—O5^v^	3.159 (2)	N5—C1	1.439 (4)
Cs2—O3^x^	3.297 (2)	N6—C3	1.453 (3)
Cs2—O3^ii^	3.297 (2)	N7—C4	1.447 (3)
Cs2—O6^ix^	3.396 (2)	N8—C6	1.432 (3)
Cs2—O6^v^	3.396 (2)	C1—C2	1.398 (3)
Cs2—N2^x^	3.401 (2)	C2—C3	1.404 (4)
Cs2—N2^ii^	3.401 (2)	C2—C5	1.458 (3)
Cs2—O2^xi^	3.811 (2)	C4—C5	1.396 (4)
Cs2—O2^vi^	3.811 (2)	C5—C6	1.399 (3)
O1—N5	1.224 (3)		
			
O1^i^—Cs1—O1^ii^	112.57 (9)	O5^ix^—Cs2—N2^x^	60.69 (6)
O1^i^—Cs1—O5^iii^	109.94 (6)	O5^v^—Cs2—N2^x^	119.31 (6)
O1^ii^—Cs1—O5^iii^	128.30 (6)	O3^x^—Cs2—N2^x^	47.39 (5)
O1^i^—Cs1—O5	128.30 (6)	O3^ii^—Cs2—N2^x^	132.61 (5)
O1^ii^—Cs1—O5	109.94 (6)	O6^ix^—Cs2—N2^x^	55.26 (5)
O5^iii^—Cs1—O5	60.14 (9)	O6^v^—Cs2—N2^x^	124.74 (5)
O1^i^—Cs1—O3^iv^	101.47 (7)	O7^viii^—Cs2—N2^ii^	119.87 (6)
O1^ii^—Cs1—O3^iv^	89.92 (7)	O7—Cs2—N2^ii^	60.13 (6)
O5^iii^—Cs1—O3^iv^	53.49 (6)	O5^ix^—Cs2—N2^ii^	119.31 (6)
O5—Cs1—O3^iv^	106.69 (6)	O5^v^—Cs2—N2^ii^	60.69 (6)
O1^i^—Cs1—O3^v^	89.92 (7)	O3^x^—Cs2—N2^ii^	132.61 (5)
O1^ii^—Cs1—O3^v^	101.47 (7)	O3^ii^—Cs2—N2^ii^	47.39 (5)
O5^iii^—Cs1—O3^v^	106.69 (6)	O6^ix^—Cs2—N2^ii^	124.74 (5)
O5—Cs1—O3^v^	53.49 (6)	O6^v^—Cs2—N2^ii^	55.26 (5)
O3^iv^—Cs1—O3^v^	159.51 (8)	N2^x^—Cs2—N2^ii^	180.0
O1^i^—Cs1—N3	151.61 (7)	O7^viii^—Cs2—O2^xi^	46.92 (5)
O1^ii^—Cs1—N3	61.45 (6)	O7—Cs2—O2^xi^	133.09 (5)
O5^iii^—Cs1—N3	92.08 (6)	O5^ix^—Cs2—O2^xi^	114.79 (5)
O5—Cs1—N3	48.49 (5)	O5^v^—Cs2—O2^xi^	65.21 (5)
O3^iv^—Cs1—N3	106.11 (6)	O3^x^—Cs2—O2^xi^	78.04 (6)
O3^v^—Cs1—N3	66.04 (6)	O3^ii^—Cs2—O2^xi^	101.96 (6)
O1^i^—Cs1—N3^iii^	61.45 (6)	O6^ix^—Cs2—O2^xi^	100.16 (5)
O1^ii^—Cs1—N3^iii^	151.61 (7)	O6^v^—Cs2—O2^xi^	79.84 (5)
O5^iii^—Cs1—N3^iii^	48.49 (5)	N2^x^—Cs2—O2^xi^	54.28 (5)
O5—Cs1—N3^iii^	92.08 (6)	N2^ii^—Cs2—O2^xi^	125.72 (5)
O3^iv^—Cs1—N3^iii^	66.03 (6)	O7^viii^—Cs2—O2^vi^	133.08 (5)
O3^v^—Cs1—N3^iii^	106.11 (6)	O7—Cs2—O2^vi^	46.91 (5)
N3—Cs1—N3^iii^	137.57 (8)	O5^ix^—Cs2—O2^vi^	65.21 (5)
O1^i^—Cs1—O4^iv^	66.05 (7)	O5^v^—Cs2—O2^vi^	114.79 (5)
O1^ii^—Cs1—O4^iv^	93.97 (7)	O3^x^—Cs2—O2^vi^	101.96 (6)
O5^iii^—Cs1—O4^iv^	77.60 (6)	O3^ii^—Cs2—O2^vi^	78.04 (6)
O5—Cs1—O4^iv^	137.70 (5)	O6^ix^—Cs2—O2^vi^	79.84 (5)
O3^iv^—Cs1—O4^iv^	36.92 (6)	O6^v^—Cs2—O2^vi^	100.16 (5)
O3^v^—Cs1—O4^iv^	155.15 (6)	N2^x^—Cs2—O2^vi^	125.72 (5)
N3—Cs1—O4^iv^	138.81 (6)	N2^ii^—Cs2—O2^vi^	54.28 (5)
N3^iii^—Cs1—O4^iv^	57.79 (6)	O2^xi^—Cs2—O2^vi^	180.00 (6)
O1^i^—Cs1—O4^v^	93.97 (7)	N5—O1—Cs1^xii^	140.2 (2)
O1^ii^—Cs1—O4^v^	66.05 (7)	N6—O3—Cs2^xii^	130.86 (18)
O5^iii^—Cs1—O4^v^	137.70 (6)	N6—O3—Cs1^xiii^	93.91 (18)
O5—Cs1—O4^v^	77.60 (6)	Cs2^xii^—O3—Cs1^xiii^	110.96 (7)
O3^iv^—Cs1—O4^v^	155.15 (6)	N6—O4—Cs1^xiii^	88.50 (16)
O3^v^—Cs1—O4^v^	36.92 (6)	N7—O5—Cs2^xiv^	99.67 (16)
N3—Cs1—O4^v^	57.79 (6)	N7—O5—Cs1	131.31 (16)
N3^iii^—Cs1—O4^v^	138.81 (6)	Cs2^xiv^—O5—Cs1	119.65 (7)
O4^iv^—Cs1—O4^v^	144.69 (7)	N7—O6—Cs2^xiv^	88.38 (14)
O1^i^—Cs1—O8^vi^	140.41 (6)	N8—O7—Cs2	118.85 (17)
O1^ii^—Cs1—O8^vi^	48.43 (6)	N8—O8—Cs1^vi^	127.02 (16)
O5^iii^—Cs1—O8^vi^	79.97 (5)	N2—N1—C1	109.8 (2)
O5—Cs1—O8^vi^	90.40 (5)	N2—N1—H1A	125.1
O3^iv^—Cs1—O8^vi^	52.64 (6)	C1—N1—H1A	125.1
O3^v^—Cs1—O8^vi^	124.92 (6)	N1—N2—C3	104.9 (2)
N3—Cs1—O8^vi^	59.05 (6)	N1—N2—Cs2^xii^	130.12 (15)
N3^iii^—Cs1—O8^vi^	116.39 (6)	C3—N2—Cs2^xii^	121.71 (17)
O4^iv^—Cs1—O8^vi^	79.82 (6)	N4—N3—C4	105.1 (2)
O4^v^—Cs1—O8^vi^	103.61 (6)	N4—N3—Cs1	128.32 (16)
O1^i^—Cs1—O8^vii^	48.43 (6)	C4—N3—Cs1	116.41 (17)
O1^ii^—Cs1—O8^vii^	140.41 (6)	N3—N4—C6	109.6 (2)
O5^iii^—Cs1—O8^vii^	90.40 (5)	N3—N4—H1B	125.2
O5—Cs1—O8^vii^	79.97 (5)	C6—N4—H1B	125.2
O3^iv^—Cs1—O8^vii^	124.92 (6)	O2—N5—O1	124.3 (3)
O3^v^—Cs1—O8^vii^	52.64 (6)	O2—N5—C1	119.1 (2)
N3—Cs1—O8^vii^	116.39 (6)	O1—N5—C1	116.6 (2)
N3^iii^—Cs1—O8^vii^	59.05 (6)	O3—N6—O4	124.1 (3)
O4^iv^—Cs1—O8^vii^	103.61 (6)	O3—N6—C3	118.4 (2)
O4^v^—Cs1—O8^vii^	79.82 (6)	O4—N6—C3	117.5 (2)
O8^vi^—Cs1—O8^vii^	168.92 (7)	O3—N6—Cs1^xiii^	66.57 (17)
O7^viii^—Cs2—O7	180.0	O4—N6—Cs1^xiii^	71.87 (15)
O7^viii^—Cs2—O5^ix^	103.32 (6)	C3—N6—Cs1^xiii^	137.60 (16)
O7—Cs2—O5^ix^	76.68 (6)	O6—N7—O5	124.3 (2)
O7^viii^—Cs2—O5^v^	76.68 (6)	O6—N7—C4	118.3 (2)
O7—Cs2—O5^v^	103.32 (6)	O5—N7—C4	117.4 (2)
O5^ix^—Cs2—O5^v^	180.00 (11)	O6—N7—Cs2^xiv^	71.61 (14)
O7^viii^—Cs2—O3^x^	106.12 (6)	O5—N7—Cs2^xiv^	60.53 (14)
O7—Cs2—O3^x^	73.88 (6)	C4—N7—Cs2^xiv^	149.11 (16)
O5^ix^—Cs2—O3^x^	54.16 (6)	O8—N8—O7	124.3 (2)
O5^v^—Cs2—O3^x^	125.84 (6)	O8—N8—C6	118.5 (2)
O7^viii^—Cs2—O3^ii^	73.88 (6)	O7—N8—C6	117.1 (2)
O7—Cs2—O3^ii^	106.12 (6)	N1—C1—C2	111.4 (2)
O5^ix^—Cs2—O3^ii^	125.84 (6)	N1—C1—N5	119.1 (2)
O5^v^—Cs2—O3^ii^	54.16 (6)	C2—C1—N5	129.5 (2)
O3^x^—Cs2—O3^ii^	180.00 (10)	C1—C2—C3	99.5 (2)
O7^viii^—Cs2—O6^ix^	68.69 (6)	C1—C2—C5	130.2 (3)
O7—Cs2—O6^ix^	111.31 (6)	C3—C2—C5	130.2 (2)
O5^ix^—Cs2—O6^ix^	38.43 (5)	N2—C3—C2	114.3 (2)
O5^v^—Cs2—O6^ix^	141.57 (5)	N2—C3—N6	117.7 (2)
O3^x^—Cs2—O6^ix^	80.81 (5)	C2—C3—N6	127.8 (2)
O3^ii^—Cs2—O6^ix^	99.19 (5)	N3—C4—C5	114.3 (2)
O7^viii^—Cs2—O6^v^	111.31 (6)	N3—C4—N7	118.3 (2)
O7—Cs2—O6^v^	68.69 (6)	C5—C4—N7	127.3 (2)
O5^ix^—Cs2—O6^v^	141.57 (5)	C4—C5—C6	99.9 (2)
O5^v^—Cs2—O6^v^	38.43 (5)	C4—C5—C2	129.6 (2)
O3^x^—Cs2—O6^v^	99.19 (5)	C6—C5—C2	130.6 (2)
O3^ii^—Cs2—O6^v^	80.81 (5)	N4—C6—C5	111.1 (2)
O6^ix^—Cs2—O6^v^	180.00 (9)	N4—C6—N8	119.0 (2)
O7^viii^—Cs2—N2^x^	60.13 (6)	C5—C6—N8	129.8 (2)
O7—Cs2—N2^x^	119.87 (6)		
			
C1—N1—N2—C3	−0.7 (3)	C1—C2—C3—N2	−0.1 (3)
C1—N1—N2—Cs2^xii^	158.78 (16)	C5—C2—C3—N2	−178.3 (2)
C4—N3—N4—C6	−0.6 (3)	C1—C2—C3—N6	175.2 (2)
Cs1—N3—N4—C6	142.57 (18)	C5—C2—C3—N6	−3.1 (4)
Cs1^xii^—O1—N5—O2	53.4 (5)	O3—N6—C3—N2	−22.2 (4)
Cs1^xii^—O1—N5—C1	−127.5 (3)	O4—N6—C3—N2	156.2 (2)
Cs2^xii^—O3—N6—O4	−167.5 (2)	Cs1^xiii^—N6—C3—N2	63.3 (3)
Cs1^xiii^—O3—N6—O4	−45.3 (3)	O3—N6—C3—C2	162.7 (3)
Cs2^xii^—O3—N6—C3	10.7 (4)	O4—N6—C3—C2	−18.9 (4)
Cs1^xiii^—O3—N6—C3	132.9 (2)	Cs1^xiii^—N6—C3—C2	−111.8 (3)
Cs2^xii^—O3—N6—Cs1^xiii^	−122.2 (2)	N4—N3—C4—C5	0.4 (3)
Cs1^xiii^—O4—N6—O3	43.4 (3)	Cs1—N3—C4—C5	−147.90 (19)
Cs1^xiii^—O4—N6—C3	−134.9 (2)	N4—N3—C4—N7	−178.0 (2)
Cs2^xiv^—O6—N7—O5	31.4 (3)	Cs1—N3—C4—N7	33.7 (3)
Cs2^xiv^—O6—N7—C4	−148.0 (2)	O6—N7—C4—N3	155.1 (3)
Cs2^xiv^—O5—N7—O6	−34.6 (3)	O5—N7—C4—N3	−24.4 (4)
Cs1—O5—N7—O6	−179.42 (19)	Cs2^xiv^—N7—C4—N3	53.4 (4)
Cs2^xiv^—O5—N7—C4	144.81 (19)	O6—N7—C4—C5	−23.1 (4)
Cs1—O5—N7—C4	0.0 (4)	O5—N7—C4—C5	157.4 (3)
Cs1—O5—N7—Cs2^xiv^	−144.8 (2)	Cs2^xiv^—N7—C4—C5	−124.8 (3)
Cs1^vi^—O8—N8—O7	26.7 (4)	N3—C4—C5—C6	−0.1 (3)
Cs1^vi^—O8—N8—C6	−152.15 (18)	N7—C4—C5—C6	178.2 (3)
Cs2—O7—N8—O8	66.2 (3)	N3—C4—C5—C2	179.4 (3)
Cs2—O7—N8—C6	−114.9 (2)	N7—C4—C5—C2	−2.3 (5)
N2—N1—C1—C2	0.7 (3)	C1—C2—C5—C4	−43.6 (4)
N2—N1—C1—N5	−178.4 (2)	C3—C2—C5—C4	134.1 (3)
O2—N5—C1—N1	169.4 (2)	C1—C2—C5—C6	135.7 (3)
O1—N5—C1—N1	−9.8 (4)	C3—C2—C5—C6	−46.5 (4)
O2—N5—C1—C2	−9.5 (4)	N3—N4—C6—C5	0.5 (3)
O1—N5—C1—C2	171.4 (3)	N3—N4—C6—N8	−176.5 (2)
N1—C1—C2—C3	−0.4 (3)	C4—C5—C6—N4	−0.3 (3)
N5—C1—C2—C3	178.5 (2)	C2—C5—C6—N4	−179.8 (3)
N1—C1—C2—C5	177.9 (2)	C4—C5—C6—N8	176.4 (3)
N5—C1—C2—C5	−3.2 (5)	C2—C5—C6—N8	−3.1 (5)
N1—N2—C3—C2	0.5 (3)	O8—N8—C6—N4	175.1 (2)
Cs2^xii^—N2—C3—C2	−161.16 (16)	O7—N8—C6—N4	−3.9 (4)
N1—N2—C3—N6	−175.3 (2)	O8—N8—C6—C5	−1.4 (4)
Cs2^xii^—N2—C3—N6	23.1 (3)	O7—N8—C6—C5	179.6 (3)

Symmetry codes: (i) −*x*+1, *y*+1, −*z*+1/2; (ii) *x*, *y*+1, *z*; (iii) −*x*+1, *y*, −*z*+1/2; (iv) *x*+1/2, *y*+1/2, *z*; (v) −*x*+1/2, *y*+1/2, −*z*+1/2; (vi) −*x*+1/2, −*y*+1/2, −*z*; (vii) *x*+1/2, −*y*+1/2, *z*+1/2; (viii) −*x*, −*y*+1, −*z*; (ix) *x*−1/2, −*y*+1/2, *z*−1/2; (x) −*x*, −*y*, −*z*; (xi) *x*−1/2, *y*+1/2, *z*; (xii) *x*, *y*−1, *z*; (xiii) *x*−1/2, *y*−1/2, *z*; (xiv) −*x*+1/2, *y*−1/2, −*z*+1/2.

### Poly[[µ4-4-(3,5-dinitropyrazol-4-yl)-3,5-dinitropyrazol-1-ido]caesium] (2) Hydrogen-bond geometry (Å, º)

**Table:**

*D*—H···*A*	*D*—H	H···*A*	*D*···*A*	*D*—H···*A*
N1—H1*A*···N4^xii^	0.87	1.99	2.832 (3)	162
N4—H1*B*···N1^ii^	0.87	1.99	2.832 (3)	163

Symmetry codes: (ii) *x*, *y*+1, *z*; (xii) *x*, *y*−1, *z*.
